# Predictive models-assisted diagnosis of AIDS-associated *Pneumocystis jirovecii* pneumonia in the emergency room, based on clinical, laboratory, and radiological data

**DOI:** 10.1038/s41598-024-61174-4

**Published:** 2024-05-16

**Authors:** Oscar José Chagas, Fabio Augusto Rodrigues Gonçalves, Priscila Paiva Nagatomo, Renata Buccheri, Vera Lucia Pereira-Chioccola, Gilda Maria Barbaro Del Negro, Gil Benard

**Affiliations:** 1 Laboratório de Micologia Médica (LIM53), Instituto de Medicina Tropical (IMT), Faculdade de Medicina (FMUSP), Universidade de São Paulo, São Paulo, SP Brazil; 2 Laboratório de Medicina Laboratorial (LIM03), Hospital das Clínicas da Faculdade de Medicina (HCFMUSP), Universidade de São Paulo, São Paulo, SP Brazil; 3 Instituto de Infectologia Emílio Ribas, São Paulo, SP Brazil; 4 Laboratório de Biologia Molecular de Parasitas e Fungos do Centro de Parasitologia e Micologia, Instituto Adolfo Lutz, São Paulo, SP Brazil; 5 Present Address: Vitalant Research Institute, San Francisco, CA USA

**Keywords:** HIV infections, Fungal infection, Diagnosis

## Abstract

We assessed predictive models (PMs) for diagnosing *Pneumocystis jirovecii* pneumonia (PCP) in AIDS patients seen in the emergency room (ER), aiming to guide empirical treatment decisions. Data from suspected PCP cases among AIDS patients were gathered prospectively at a reference hospital's ER, with diagnoses later confirmed through sputum PCR analysis. We compared clinical, laboratory, and radiological data between PCP and non-PCP groups, using the Boruta algorithm to confirm significant differences. We evaluated ten PMs tailored for various ERs resource levels to diagnose PCP. Four scenarios were created, two based on X-ray findings (diffuse interstitial infiltrate) and two on CT scans (“ground-glass”), incorporating mandatory variables: lactate dehydrogenase, O2_sat_, C-reactive protein, respiratory rate (> 24 bpm), and dry cough. We also assessed HIV viral load and CD4 cell count. Among the 86 patients in the study, each model considered either 6 or 8 parameters, depending on the scenario. Many models performed well, with accuracy, precision, recall, and AUC scores > 0.8. Notably, nearest neighbor and naïve Bayes excelled (scores > 0.9) in specific scenarios. Surprisingly, HIV viral load and CD4 cell count did not improve model performance. In conclusion, ER-based PMs using readily available data can significantly aid PCP treatment decisions in AIDS patients.

## Introduction

Global HIV data show that in 2021, 38,4 million people were living with HIV (PLHIV) worldwide, with 650,000 associated deaths. Most of these deaths occurred in Sub-Saharan Africa, followed by East Asia and Latin America^1,2^. In Brazil, recent data reported 50,000 new annual infections, a 5% increase since 2010, and almost 13,000 associated deaths^3^. Unfortunately, late presentation to care and initiation of antiretroviral therapy (ART) with advanced HIV disease are still common in Latin America, with almost 56% of the new diagnoses having T CD4 lymphocytes (CD4) counts below 200 cells/mm^3^ at the time of diagnosis^2,4^. Consequently, opportunistic infections remain a major cause of HIV-associated deaths in this region^5,6^.

Although *Pneumocystis jirovecii* pneumonia (PCP) incidence has continuously decreased after the introduction of ART and prophylaxis^7,8^, it remains among the leading pulmonary opportunistic infections in several developing and developed countries^5,6^. The estimated incidence in Brazilian AIDS patients varies widely, ranging from 5.6 to 36%, owing to the variability in the methods and source of samples used to reach the diagnosis^9,10^. PCP accounts for almost 400,000 cases/year, with 200,000 deaths/year, mainly in developing countries^11^.

Diagnosing PCP continues to pose challenges due to various factors, including the lack of conventional culture systems for *P. jirovecii*^12^, the limited specificity of clinical symptoms, the reduced sensitivity of the usual diagnostic methods, and the complexities associated with sample collection. Numerous studies have highlighted the polymerase chain reaction (PCR) assay as a more sensitive method for diagnosing PCP. However, no standard technique has been widely incorporated in routine laboratories, nor are molecular biology and biomarkers assays easily accessible^13^. As a result, the lack of a PCP diagnosis leads to the implementation of empirical treatment in almost all cases, particularly in resource-limited settings.

More recently, the expanded use of machine learning (ML) has increased the possibilities of using health care data, enabling the creation of systems that assist human decision^14^. ML has already been tested in different areas of health care, showing promising clinical applications^15^. Several reports of ML application in infectious diseases improved the diagnosis, especially in settings lacking specific laboratory or radiology tests^16^.

Our research aimed to identify and evaluate predictors associated with PCP in AIDS patients among different types of supervised ML algorithms. We constructed predictive models based on clinical, laboratory, and radiology aspects easily accessible at most emergency rooms (ERs), including those from low-income countries. Some of the predictive models achieved high accuracy in different ERs’ scenarios. They can constitute valuable tools to improve the physicians' decision-making process of treating AIDS patients with suspected PCP.

## Material and methods

### Study design and patients

This was a prospective study that enrolled AIDS patients admitted between December 2016 and February 2020 at the ER of the Instituto de Infectologia Emílio Ribas (IIER), who were initially suspected of having PCP according to the following criteria: the presence of subacute cough and dyspnea (≥ 7 days), a current CD4 cell count < 250 cells/mm^3^, and poor compliance to or not on ART. Induced sputum was collected in a room with negative pressure before starting treatment for PCP (or with up to one dose) through inhalation of hypersaline solution (3–5% of NaCl), for 15–20 min, collected in a sterile container and stored at 4ºC until DNA extraction up to the next day, as previously described^17^. We performed an “in-house” quantitative PCR (qPCR) assay after DNA extraction of induced sputum, and serum samples collected simultaneously to the induced sputum were tested with the Fungitell® assay^18^ (Associates of Cape Cod, East Falmouth, MA, USA) for (1,3)-β-d-glucan (BDG) measurement according to the manufacturer's instructions.

We used this qPCR as standard diagnoses and considered patients with PCP when the threshold (Cq) of the qPCR was less or equal to 31 and colonized or without PCP when Ct was greater than 31, as previously described^17^. We collected demographic, clinical, laboratory, and radiological data of all patients. To predict PCP, we opted to include data usually associated with PCP in AIDS patients, which could be quickly accessed at ERs with different levels of resources (Table 1).

**Table 1:** Characteristics that were statistically significant between the group with PCP (Cq ≤ 31) and without PCP (Cq > 31).

Characteristics	Non PCP (n = 54)^a^	PCP (n = 32)^a^	Total	p-value^b^
Presence of dry cough	13/54 (24%)	18/32 (56%)	31/86 (36%)	0.005
Respiratory frequency (bim)	24 (20–30)	28 (24–35)	26 (20–32)	0.02
Respiratory frequency (> 24 bim)	24/54 (44%)	5/32 (16%)	29/86 (34%)	0.009
O_2_ saturation (%) – pulse oximetry	94 (92–96)	91.5 (85.8–95)	94 (90–96)	0.01
X-ray with diffuse interstitial infiltrate	33/54 (41%)	30/32 (94%)	63/86 (73%)	< 0.001
CT scan with "ground grass"	22/54 (41%)	30/32 (94%)	52/86 (60%)	< 0.001
LDH (U/L)	248 (200–388)	422 (319–569	302(223–496)	< 0.001
CRP (mg/dL)	134. (67–199)	63 (34–107	96 (43–186)	0.019
O_2_ saturation (%)—arterial blood gas	95 (93–96)	92 (88–95)	94 (92–96)	0.005
CD4 cell count (cells/mm^3^)	58.5 (26.8–106.1)	15.5 (6.8–54.8)	38.5 (10–92)	0.003
HIV viral load (copies/mL)	90,787 (6,254–262,203)	324,527 (72,531–860,093)	142,435 (20,312- 442,627)	0.002
CMV disseminated disease	5/54 (9.3%)	9/32 (28%)	14/86 (16%)	0.033
BDG (pg/mL)	26 (1–69)	523 (349–523)	71 (10–523)	< 0.001
O_2_ saturation > 94% (pulse oximetry)	25/54 (46%)	10/32 (31%)	35/86 (41%)	0.2

^a^n/N (%); Median (IQR).

^b^Fisher´s exact test; Wilcoxon rank sum test.

### Statistical analysis

All categorical variables were compared using Fisher's exact test, and continuous variables were tested for normal distribution using the Shapiro–Wilk test before statistical analysis. The Shapiro–Wilk test showed a non-normal distribution of all variables. The continuous variables were expressed as the median and interquartile range (IQR) and compared using the Student t test.

**Figure 1:**
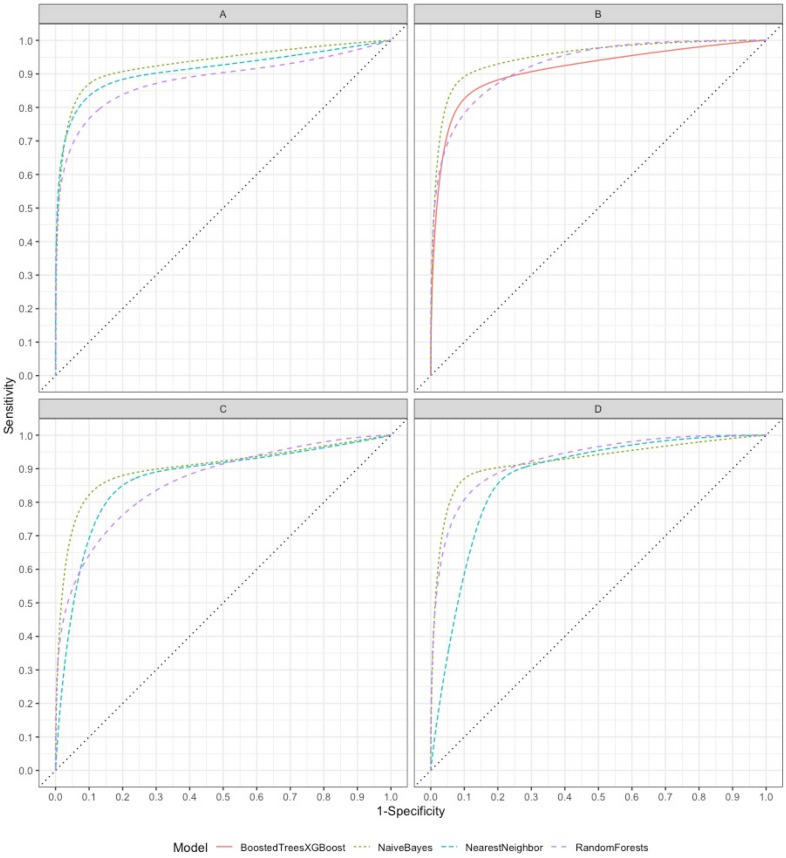
Area under the curve (AUC) of the predictive models with best performance calculated for each of the A, B, C and D scenarios: extreme gradient boosting (XGboost), Naïve Bayes, nearest neighbor, and random forest. Figure 1 shows AUC from predictive models that presented a greater performance for each scenario. Scenario A: NN, NB and RF. Scenario B: NB, RF, and XGBoost. Scenario C: NB, RF and NN. Scenario D: RF, NB and NN.

The patients' variables that were gathered were first tested by classical statistical models comparing the patients with qPCR-confirmed PCP with those in whom the qPCR ruled out PCP. The variables that presented statistical difference were additionally evaluated through Boruta algorithm (Fig. 1—Supplementary information)^19^. The validated variables were further analyzed using univariable and multivariable logistic regression to calculate the odds ratio (OR) and corresponding 95% confidence interval (CI) to confirm whether the selected variables are risk factors for PCP before being considered for use in the predictive models. All statistical analyses were performed using R Statistical Software v4.2.2 (R Core Team, 2022: A language and environment for statistical computing, R Foundation for Statistical Computing, Vienna, Austria)^20^. For all analyses, differences with *p* < 0.05 were deemed statistically significant.

### Data preprocessing

Before model fitting, categorical variables were transformed into binary dummy variables, as most predictive models are affected by the difference in the variables' scales. As data contained various scales for various quantities (e.g., C-reactive protein (CRP), lactate dehydrogenase (LDH), CD4 cell count, HIV viral load), data normalization was necessary to rescale all numeric values with a standard deviation of one and a mean of zero. This makes the various predictive models more effective. All values were normalized to reduce the dimension-introduced bias using Z-score standardization^21^. The dataset was randomly divided into a 70% training set to construct the predictive model and a 30% testing set for performance assessment, stratifying by the PCP outcome^22^.

#### Missing values

For physical parameters, radiological and laboratory data, which were associated with observed variables based on the clinical decision practice, we identified missing, not at random. The overall dataset exhibited a missing data rate of 3%. For each variable requiring imputation, a bagged tree was created where the outcome is the PCP variable, and the predictors are all other variables. One advantage of the bagged tree is that it can accept predictors with missing values^23^. The matrix layout of all intersections is demonstrated in the supplementary material (Fig. 2—Supplementary information).

#### Imbalanced data

This dataset was unbalanced. In this study, an unbalanced ratio showed that the minority class was 51.2%, less than the majority class when analyzing the number of observations. Therefore, to reduce data bias, we opted for the synthetic minority over-sampling technique (SMOTE)^24^, which manages overfitting induced by a limited decision interval and controls the generation and distribution of manual samples using the minority class sample.

#### Predictive models

Predictive models training may overfit algorithms to the nuances of a specific dataset, resulting in a model that does not generalize well to new data^22^. We compared ten predictive models to evaluate their effectiveness in predicting PCP in patients with AIDS. For the linear model, we opted for simple probabilistic classifiers, such as Naïve Bayes (NB)^25^, elastic network model (EN)^26^, and linear support vector machines (LSVM)^27^. For the kernel-based model, we utilized a multilayer perceptron (MLP)^28^. For the decision tree approach, the random forest (RF) model^29^, decision tree, bagged trees (BT), boosted trees light GBM (LightGBM), and the extreme gradient boosting (XGBoost) model^30^ have been used. Finally, multi-class algorithms as nearest neighbor (NN) were built^31^. We aimed to include different classes of ML methods.

#### Evaluation metrics

In the training set, the k-fold cross-validation with three folds and ten resamples was used to mitigate the potential bias or variance issues stemming from a single train-test split. An ANOVA-based racing tuning method was employed to optimize the hyperparameters for each candidate model, focusing on accuracy enhancement^32^.

Finally, after completing adjustments and training with the training set, the models were evaluated against the test set to ensure an accurate estimation of the performance of the model candidates without overfitting. The accuracy, precision, recall, F1-Score, and the area under the ROC curve (AUC) of each model were evaluated to establish a model ranking. Generally, these metrics indicate good performance when scores exceed 0.8 and poor performance below 0.7^33^.

### Ethical approval

The Comitê de Ética em Pesquisa from the Instituto de Infectologia Emílio Ribas approved the study (protocol 06/2016). All study was conducted in accordance with relevant institutional guidelines, and all patients consented to participate by signing an informed consent form.

## Results

Ninety-seven PLHIV admitted to the emergency unit of the IIER with respiratory manifestations suggestive of PCP were enrolled. Eight patients were excluded for being transferred to another health service within the first 24 h of admission (n = 6) or for failing to provide induced sputum (n = 2). Therefore, 86 patients underwent the radiology and laboratory workouts prescribed by the attending physician. Variables statistically different between the two groups, with and without qPCR-proven PCP, are shown in Table 1. Additional sociodemographic and clinical data are shown in Supplementary Table 1. Patients with PCR results suggestive of colonization were grouped with the PCR negative patients, since the purpose of the study was to support the treatment decision.

As previously described, the two groups did not significantly differ regarding sociodemographic aspects or other clinical, radiology, and laboratory variables^17^.

In our study, the clinical, laboratory, and radiological variables commonly associated with PCP that showed statistical differences were as follows: dry cough, increased respiratory frequency, decreased O_2_ saturation (O2_sat_) in arterial blood gas, elevated LDH levels, lower CRP values, low CD4 cell count, higher HIV viral load, chest X-ray showing diffuse interstitial infiltrate (DII), CT scan indicating a “ground-glass” image, presence of associated cytomegalovirus disease (CMV), and higher BDG values. BDG value was excluded since it is not available in most Brazilian ERs. These variables were then submitted to Boruta's analyses to determine the weight of each to the diagnosis of PCP. Boruta's analysis validated all variables except CMV co-infection. Ground-glass opacity on the CT scan was most strongly associated with PCP prediction, followed by LDH, arterial O2_sat_, CRP, and HIV viral load. Less but still significantly associated with PCP prediction were chest X-ray with DII, CD4 cell count, a respiratory rate greater than 24 bpm, and dry cough (Fig. 1—Supplementary information).

In parallel, we also designed four possible scenarios aiming at encompassing the variable range of facilities provided at ERs in Brazil, as depicted in Table 2. We used six variables in two scenarios and eight variables in the other two. The scenarios were headed depending on whether the ER has X-ray equipment or a CT scan (which presents greater sensitivity for diagnosing interstitial pulmonary diseases^34^), associated with the following set of variables: LDH (U/L), O2_sat_ on arterial blood (%), CRP (mg/dL), respiratory rate > 24 bpm and dry cough. As CD4 cell and HIV viral load are carried out only in a few Brazilian Ministry of Health's reference laboratories, their results are not promptly accessible, so they were included for analyses only in secondary scenarios as additional variables.

**Table 2:** Features of Brazil's ERs: four possible scenarios.

Scenario A: Chest X-ray + Mandatory variables^a^
Scenario B: Thorax CT scan + Mandatory variables
Scenario C: Chest X-ray + Mandatory variables + Additional variables^b^
Scenario D: Thorax CT scan + Mandatory variables + Additional variables

^a^Mandatory variables: LDH (U/L)/SatO_2_ on arterial blood (%)/CRP (mg/dL)/respiratory rate > 24 bpm/dry cough.

^b^Additional variables: HIV viral load (copies/mL)/CD4 cell counts (cells/mm^3^).

We applied ten predictive models, as described in the methods section, to the four scenarios and used five metrics to evaluate the designed models' performance, as presented in Tables 3, 4, 5, and 6. Recall is relevant in settings where no patient should miss specific treatment because, e.g., the disease may be life-threatening (as is the case in PCP). However, it can otherwise lead to the treatment of false positive cases. Precision informs the capacity of the model to indicate the correct treatment for true positive PCP cases. Accuracy corresponds to both the ability to implement treatment for truly positive PCP cases and not implementing treatment for negative patients. AUC indicates the utility of the predictor in giving the best points of balance between true positive and false positive rates and summarizing the performance across all operating point tradeoffs.

**Table 3:** (Scenario A): Performance of predictive models for Scenario A (Chest X-ray with DII + mandatory variables: LDH (U/L)/O2_sat_ on arterial blood (%)/CRP (mg/dL)/respiratory rate > 24 bpm/dry cough).

Model	Accuracy	Precision	Recall	F1-Score	AUC
NearestNeighbor	0.923	0.900	0.9	0.900	0.909
RandomForests	0.885	1.000	0.7	0.824	0.906
NaiveBayes	0.885	0.818	0.9	0.857	0.963
ElasticNet	0.808	0.778	0.7	0.737	0.800
DecisionTree	0.769	0.833	0.5	0.625	0.712
BoostedTreesLightGBM	0.731	0.714	0.5	0.588	0.728
LinearSVM	0.731	0.667	0.6	0.632	0.737
BaggedTrees	0.692	0.600	0.6	0.600	0.766
BoostedTreesXGBoost	0.692	0.625	0.5	0.556	0.763
MultilayerPerceptron	0.692	0.600	0.6	0.600	0.656

**Table 4:** (Scenario B): Performance of predictive models for Scenario B (Thorax CT scan with "ground-grass" opacity + mandatory variables: LDH (U/L)/O2_sat_ on arterial blood (%)/CRP (mg/dL)/respiratory rate > 24 bpm/dry cough).

Model	Accuracy	Precision	Recall	F1-Score	AUC
NaiveBayes	0.923	0.900	0.9	0.900	0.981
RandomForests	0.885	0.818	0.9	0.857	0.969
BoostedTreesXGBoost	0.885	0.889	0.8	0.842	0.938
BaggedTrees	0.846	0.875	0.7	0.778	0.934
NearestNeighbor	0.846	0.750	0.9	0.818	0.950
DecisionTree	0.808	0.727	0.8	0.762	0.741
BoostedTreesLightGBM	0.808	0.727	0.8	0.762	0.906
MultilayerPerceptron	0.808	1.000	0.5	0.667	0.812
ElasticNet	0.769	0.643	0.9	0.750	0.925
LinearSVM	0.769	0.643	0.9	0.750	0.881

**Table 5:** (Scenario C): Performance of the predictive models for Scenario C (Chest X-ray with DII + mandatory variables: LDH (U/L)/O2_sat_ on arterial blood (%)/CRP (mg/dL)/respiratory rate > 24 bpm/dry cough + additional variables: HIV viral load (copies/mL)/CD4 cell counts (cells/mm^3^)).

Model	Accuracy	Precision	Recall	F1-Score	AUC
NaiveBayes	0.885	0.818	0.9	0.857	0.925
RandomForests	0.846	0.750	0.9	0.818	0.913
NearestNeighbor	0.846	0.750	0.9	0.818	0.900
LinearSVM	0.846	0.750	0.9	0.818	0.888
BoostedTreesXGBoost	0.769	0.643	0.9	0.750	0.813
BaggedTrees	0.731	0.636	0.7	0.667	0.828
ElasticNet	0.654	0.538	0.7	0.609	0.769
DecisionTree	0.654	0.538	0.7	0.609	0.759
BoostedTreesLightGBM	0.654	0.538	0.7	0.609	0.763
MultilayerPerceptron	0.654	0.538	0.7	0.609	0.787

**Table 6:** (Scenario D): Performance of predictive for Scenario D (Thorax CT scan with “ground-grass” opacity + mandatory variables: LDH (U/L)/O2_sat_ on arterial blood (%)/CRP (mg/dL)/respiratory rate > 24 bpm/dry cough + additional variables: HIV viral load (copies/mL)/CD4 cell counts (cells/mm^3^)).

Model	Accuracy	Precision	Recall	F1-Score	AUC
RandomForests	0.923	0.900	0.9	0.900	0.950
NaiveBayes	0.885	0.818	0.9	0.857	0.944
DecisionTree	0.846	0.714	1.0	0.833	0.875
BaggedTrees	0.846	0.800	0.8	0.800	0.866
NearestNeighbor	0.846	0.750	0.9	0.818	0.938
LinearSVM	0.846	0.750	0.9	0.818	0.900
ElasticNet	0.808	0.692	0.9	0.783	0.900
BoostedTreesXGBoost	0.808	0.692	0.9	0.783	0.938
BoostedTreesLightGBM	0.769	0.643	0.9	0.750	0.888
MultilayerPerceptron	0.654	0.533	0.8	0.640	0.831

All ten models performed satisfactorily in the four scenarios, suggesting that selecting the variables based on prior knowledge of statistical and Boruta analyses was appropriate. Four notably performed particularly well: NB, NN, RF, and XGBoost. They in general yielded indices greater than 0.8 for most scenarios and all five metrics, which is the usual recommendation for diagnostic tests^33^. One of the most familiar criteria used in the literature to evaluate the performance of a predictive model is the AUC, whose overall performance allows us to compare the performance of the predictive models graphically. Figure 1 depicts the AUC for these four models in the four scenarios, showing frequent indices above 0.9. However, as our primary goal is to provide treatment only for true PCP cases, avoiding unnecessary treatment of non-PCP cases, we opted for accuracy as the major criterion. Accuracy measures the overall correctness for true positive and true negative patients, informing the ability to implement treatment for PCP and not for non-PCP patients. Furthermore, accuracy, precision, and negative predictive value are prevalence-dependent metrics, whereas AUC, recall, and specificity are prevalence-independent.

Concerning the scenario A (Table 3), which mimics the usual common ERs’ setting (i.e., an X-ray is available, but not a CT scan), the NN model yielded the highest accuracy score (0.923), followed closely by both RF and NB with 0.885. All three also showed an AUC > 0.9. NN and NB presented precision and recall indices > 0.8. Although the RF model reached the highest precision (1.0), it presented a low recall (0.7), negatively impacting its F1-score. In addition, a fourth model, EN, also showed high accuracy (> 0.8) but somewhat weaker precision (0.78) and recall (0.7) scores. The remaining six models performed modestly only compared to those above three yielded accuracy indices between 0.7 and 0.8 and three below 0.7, with variable performances below 0.8 in the other criteria.

In scenario B (Table 4), the models using CT scan instead of X-ray showed overall better performances than in scenario A, considering the remarkable (n = 8) number of predictive models that reached accuracy values > 0.8. This is likely because the thoracic CT scan has greater sensitivity than chest X-rays in detecting pulmonary interstitial lesions^35^. Differently from scenario A, in scenario B it was the NB that reached the highest accuracy (0.923) as well as ≥ 0.9 scores in the other metrics, especially the AUC, with a score of 0.981. Additional seven predictive models presented high accuracy scores (≥ 0.8), such as RF and XGBoost (0.885), with high scores (≥ 0.8) also in the other metrics. Although BT and NN showed good accuracy (0.846), NN yielded a modest precision (0.75), and BG a modest recall score (0.7). The remaining five models, decision tree, LightGBM, MP, EN, and LSVM, performed somewhat more modestly than those mentioned above.

The analyses of scenarios, including thorax CT scan, raised the issue of how important this variable for the models' performance is. Even though its recognized better performance for diagnosing interstitial diseases, in scenarios B and D the models reached scores like those with chest X-ray, except for the highest AUC of 0.981 with the NB in scenario B. The presence of “ground-glass” opacity in the thorax CT scan of PLHIV presenting pulmonary symptoms is well-established as highly associated with PCP or viral infections^35^. However, it is not a specific signal and should not be taken alone for diagnosing PCP, especially in AIDS patients who not uncommonly develop concomitant pulmonary opportunistic infections^35^. For this reason, we still recommend its utilization in settings where a CT scan is available.

In scenario C (Table 5), unexpectedly, adding CD4 cell count and HIV viral load to the variables of scenario A did not result in higher performances, with the highest accuracy score being 0.885 (NB). Four models reached an accuracy greater than 0.8, with recalls of 0.9. Still, three of them had precision values < 0.8, which can lead to the undesired outcome of implementing empirical treatment in non-PCP patients. Overall, the models' performance in this scenario was slightly weaker than in scenarios A and B.

Scenario D (Table 6), with the addition of CD4 cell count and HIV viral load to the set of variables, also did not further improve the model's accuracy. The highest accuracy score was reached with RF (0.923), which also yielded scores greater than 0.9 regarding precision, recall, and AUC, a performance much like that observed with the NB in scenario B. In scenario B, the other seven models presented accuracy scores > 0.8. NB reached the second-highest best accuracy (0.885), followed closely by decision tree, BT, NN, and LSVM (0.846). These four models also performed well in the other metrics, reaching values ≥ 0.8.

## Discussion

Predictive models for diagnostic purposes have already been tested in different areas of health care^36^. Although many specialties were covered^36^, there has been special interest in evaluating predictive models to improve decision-making processes in infectious diseases, from diagnosis to the risk of developing symptomatic infection and from predicting severity/mortality or complications to treatment response. These studies applied a wide range of models, the most commonly used being support vector machine (SVM), XGBoost, decision tree, RF, and NB, and several metrics used in the present study^36^. Of the ten models we have tested, NB, RF, and NN presented the overall best performance, with NB being increasingly studied and generally yielding good accuracy results^37^.

The use of predictive models in infectious diseases can be exemplified by the numerous models tested as alternative methods to diagnosing SARS-CoV-2 infection in a period when laboratory diagnosis was a challenge due to the high volume of patients, among other issues^38^. For example, Mei et al. 2020, evaluated a data set acquired from Chinese patients for whom there was a clinical concern of COVID-19 between January and March 2020. SVM, RF, and MLP were applied using pulmonary CT scan data associated with easily accessible demographic, clinical, and laboratory variables similar to our study. Confirmatory diagnosis of COVID-19 infection was achieved by real-time PCR (RT-PCR), being positive in 46.9% of the cohort. In this study, MLP performed better than the other two models, reaching a sensitivity of 0.843, a specificity of 0.828, and an AUC of 0.92. However, contrary to our study, where imaging evaluation was based on the presence/absence of interstitial infiltrate/ground grass images according to the ER clinicians' interpretation, they used a convolutional neural network model for CT scan analyses, which limits its applicability to limited-resource ERs^39^. In addition, our slightly better results could be accounted for, at least in part, by using Boruta's analysis of selected PCP-associated variables. This step seems important to increase the performance and can bring more confidence and adhesion by the clinicians than using random variables. We also designed our study to test a larger number of models to find the one that provided the best fit.

Predictive models were also used to investigate other viral diseases with some diagnostic challenges^40^. Dengue diagnosis was retrospectively studied in a cohort of Paraguayan patients with fever and initial clinical dengue suspicion, subsequently confirmed either by IgM serology, virologic isolation, or RT-PCR. Moreover, the authors used the SVM, MLP, and radial basis function as predictive models throughout 37 clinical-epidemiological and demographic variables that can be associated with dengue. SVM performed better, reaching an accuracy of 0.92 as well as a sensitivity of 0.93 and specificity of 0.92, providing an apparently helpful tool for the viral infection diagnosis^40^.

Studies comparable to ours were also done in acute bacterial diseases but with less successful results. A study investigated several models in diagnosing *Clostridioides difficile* infection (CDI) in a cohort of inpatients undergoing *C. difficile* testing. This study used clinical-demographic and laboratory data and, as our study, ten different predictive models. However, all 10 presented weak performances, with AUC up to 0.60 (the single metric used). In addition, classical CDI-associated parameters were chosen, such as high white blood cells and creatinine value, which did not improve the performance. One possible concern is the eventual gastrointestinal tract colonization with *C. difficile*, which can confound the diagnosis: in this study, from 3514 possible CDI records, only 136 were confirmed^41^.

The use of predictive models to study invasive fungal infections is still rare despite the fact that diagnosis of such infections still poses a challenge: usual diagnostic methods (e.g., blood culture) exhibit low sensitivity (compared with other types of infectious agents), some fungi lack or have slow growing properties in culture media, and in several instances, differentiation between colonization and invasion is difficult^42^. A review of ML methods applied to clinical microbiology found 97 valid articles; only three dealt with fungal infections^16^. Ripoli et al. 2020, evaluated a model to predict candidemia bloodstream infection (CBI) in at-risk patients using the records of a cohort of 157 patients with confirmed candidemia (positive blood culture) compared to 138 patients with bacteremia. The RF was applied to 17 clinic-demographic variables associated with an increased risk of developing candidemia. This model reached an AUC of 0.87, a sensitivity of 0.84, and a specificity of 0.91^43^. As in the present study, the model's good performance was likely linked to the appropriate selection of variables. However, using blood culture as a gold standard may misdiagnose some patients, especially those with low fungal burden. These promising results warrant that validation studies or other prospective real-world studies are undertaken. Another recently published study applied predictive models similar to ours in the context of PCP in kidney transplant recipients, with good results. However, the focus was not on the diagnosis of PCP but on the design of a prognostic model to predict the development of severe disease following PCP in these patients^44^.

In fact, one major concern in ML studies aiming to improve medical processes is that there is little evidence that these models have entered into clinical practice. External validation is a mandatory step since assessing the model's reproducibility and generalization is fundamental. Predictive models should not be addressed before extensive evaluation since mistakes and patient harm can occur, which enhances the importance of clinical knowledge and judgment. However, a survey of PubMed using "prediction models" retrieved almost 90,000 related articles in the year 2019, but when searched allied with "external validation," only 7% of the studies remained^45^.

Although we are just beginning to understand the wealth of opportunities afforded by ML methods, there is a growing concern in the academic community that, because the products of these methods are not perceived in the same way as other medical interventions, they do not have well-defined guidelines for development and use, and rarely undergo the same degree of scrutiny as others new technologies. The kind of evidence necessary to adequately recommend the widespread use of ML methods is still debated^46^. Some steps should be followed to build confidence in the prediction model, such as adequate reporting of data source, study design, modeling processes, number of predictors, etc., which facilitates the interpretation and increases the clinician's confidence. Predictive models are not meant to replace a clinician's judgment, and they should be tested through application within existing workflows to convince clinicians of the test's applicability since they tend to resist processes that interfere with their routine or challenge their autonomy^47,48^.

Our study was conducted at the emergence room of a teaching reference center for infectious diseases, where the clinicians are highly skilled in diagnosing and treating AIDS-associated OIs. Empiric treatment was prescribed to 90% of the cohort's patients who subsequently confirmed the diagnosis of PCP, but also to 30% of the patients in whom PCP was later ruled out (data not shown). On the other hand, the NN (scenario A) and NB (scenario B) predictive models would also indicate treatment for 90% of the confirmed PCP patients while treating only 1 out of 16 (6.25%) non-PCP patients, even if used by inexperienced clinicians. Unexpectedly, including CD4 cell count and HIV viral load did not improve overall predictive models' performances (Table C and D), suggesting that, in our setting, they functioned only as marginal predictors. A likely explanation relies on the patients' inclusion criterium of absence or irregular use of ART. Almost all (95%) of the patients had comparable high HIV viral load, and all had comparable low CD4 cell count (< 250 CD4 cells/mm).

Conversely, we estimate that implementing our tested model in non-specialized infectious diseases ERs may bring even more substantial improvement in the empirical treatment of patients with presumed PCP. We plan to proceed with validation studies at our reference hospital and other ER settings where patients with PCP are less prevalent and the medical staff is not specially trained in PCP diagnosis. Other limitations of our study are the relatively small sample size of the cohort and the fact that the data source arose from a single, reference hospital for infectious diseases with a high burden of AIDS patients, making it important cross-validation studies with larger cohorts.

## Conclusion

In conclusion, after testing scenarios mimicking different ER settings, representative of either low/middle or wealthy countries, we strongly recommend that validation studies to be conducted with NN in X-ray-equipped ERs and with NB for CT scan-equipped ERs. Our models could be easily implemented in ER routine protocols to aid clinicians, particularly those not skilled in HIV/AIDS opportunistic infections, in the decision of introducing (or not) empirical treatment for suspected PCP patients.

### Supplementary Information

Supplementary Information 1.

Supplementary Information 2.

Supplementary Information 3.

## Data Availability

The data used in this study are available from the corresponding author upon reasonable request.

## Abbreviations

ART: Antiretroviral therapy
AUC: Area under the receiver operating characteristic (ROC) curve
BDG: (1-3)-β-B-glucan
CD4: Lymphocytes T CD4
CMV: Cytomegalovirus disease
CRP: C-reactive protein
CT: Computerized tomography
DII: Diffuse interstitial infiltrate
ER: Emergency room
LDH: Lactate dehydrogenase
ML: Machine learning
PCP: *Pneumocystis jirovecii* Pneumonia
PCR: Polymerase chain reaction
PLHIV: People living with HIV
