# Supplementary material for: Predictive models-assisted diagnosis of AIDS-associated Pneumocystis jirovecii pneumonia in the emergency room, based on clinical, laboratory, and radiological data
Source: Sci Rep. 2024 May 16;14:11247. doi: 10.1038/s41598-024-61174-4 (PMC11099134; doi:10.1038/s41598-024-61174-4)
Supplement: Supplementary file 1 — Supplementary Information 1. [file 41598_2024_61174_MOESM1_ESM.docx]

| **Supplementary Figure S1:** Feature selection based on the Boruta algorithm. |
| --- |
| 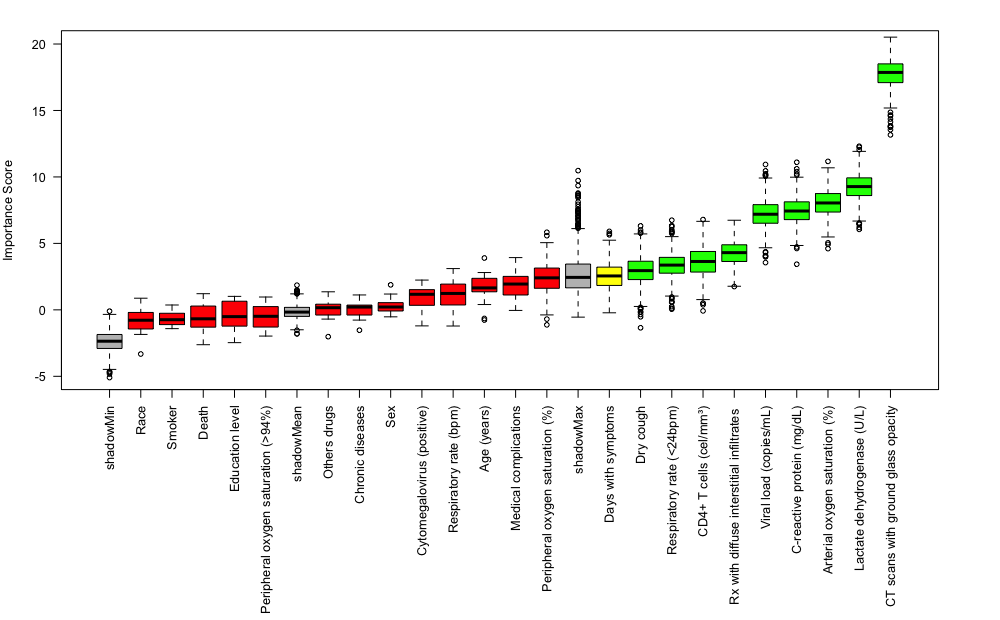 |
| **Note:** The horizontal axis is the name of each variable, and the vertical axis is the Z-value of each variable. The boxplot shows the Z-value of each variable during model calculation. The green boxes represent the ten important variables, and the red represents unimportant variables. |
