# Supplementary material for: Predictive models-assisted diagnosis of AIDS-associated Pneumocystis jirovecii pneumonia in the emergency room, based on clinical, laboratory, and radiological data
Source: Sci Rep. 2024 May 16;14:11247. doi: 10.1038/s41598-024-61174-4 (PMC11099134; doi:10.1038/s41598-024-61174-4)
Supplement: Supplementary file 2 — Supplementary Information 2. [file 41598_2024_61174_MOESM2_ESM.docx]

| **Figure 2:** Matrix layout for all intersections of the five predictors with missing data, sorted by frequency, in patients with or without PCP. |
| --- |
| 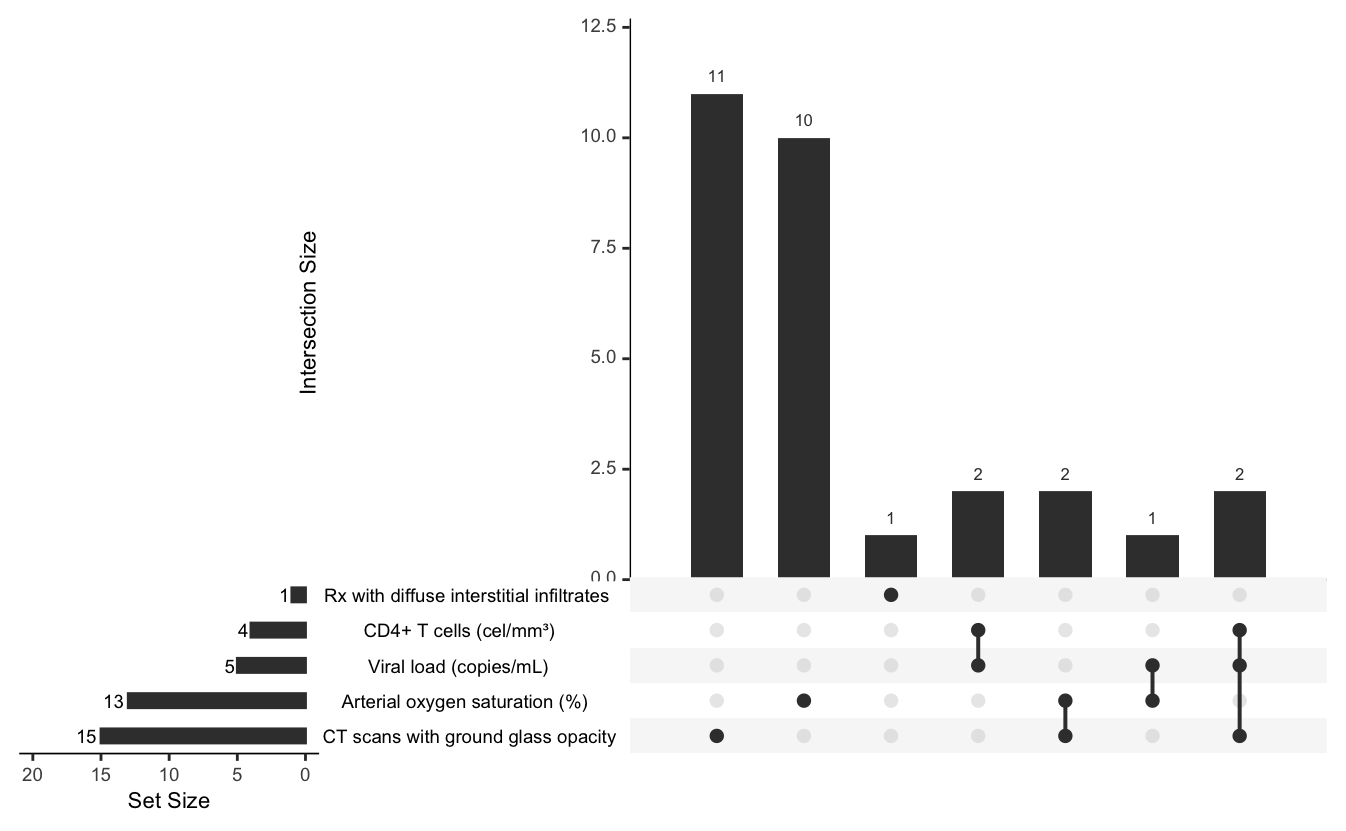 |
| **Note:** The overall dataset exhibited a missing data rate of 3% and the subset patients with or without PCP showed a rate of 0.9% and 5%, respectively. |
