# Supplementary material for: Predictive models-assisted diagnosis of AIDS-associated Pneumocystis jirovecii pneumonia in the emergency room, based on clinical, laboratory, and radiological data
Source: Sci Rep. 2024 May 16;14:11247. doi: 10.1038/s41598-024-61174-4 (PMC11099134; doi:10.1038/s41598-024-61174-4)
Supplement: Supplementary file 3 — Supplementary Information 3. [file 41598_2024_61174_MOESM3_ESM.docx]

| Supplementary Table 1: Characteristics that were not statistically significant between the group with PCP (Cq ≤ 31) and without PCP (Cq > 31). | | | | |
| --- | --- | --- | --- | --- |
| Characteristics | | **Non PCP (n = 54) ¹** | **PCP (n = 32) ¹** | **p-value ²** |
| Qualitative data | |  |  |  |
| Sex | Male | 39/54 (72%) | 26/32 (81%) | 0.3 |
|  | Female | 15/54 (28%) | 6/32 (19%) |  |
| Other chronic diseases (hypertension, diabetes) | | 9/51 (17%) | 2/31 (6%) | 0.26 |
| Smoking | Yes | 30/53 (57%) | 12/31 (39%) | 0.17 |
|  | No | 23/53 (43%) | 19/31 (61%) |  |
| Concomitant opportunistic infections | | 39/54 (72%) | 25/32 (78%) | 0.72 |
| Oral/esophageal candidiasis | | 29/54 (54%) | 20/32 (62%) | 0.56 |
| Tuberculosis | | 25/54 (46%) | 6/32 (19%) | 0.22 |
| Cytomegalovirus infections | | 5/54 (9%) | 9/32 (28%) | 0.04 |
| Toxoplasmosis | | 1/54 (2%) | 2/32 (6%) | 0.64 |
| Cryptococcosis | | 2/54 (4%) | 1/32 (3%) | 1 |
| Quantitative data | | **Non PCP (n = 54) ¹** | **PCP (n = 32) ¹** | **p-value ³** |
| Age (years) | | 41.8 ± 11.3 | 37.6 ± 9.2 | 0.21 |
| Days of symptoms | | 43.0 ± 39 | 43.4 ± 44 | 0.9 |
| HIV infection (years) | | 11.7 ± 8.8 | 7.9 ±7.8 | 0.03 |
| ¹ n/N (%); Mean ± standard deviation  ² Fischer´s exact test  ³ Student t test | | | | |
